# Genito-Urinary and Syphilitic Diseases

**Published:** 1896-08

**Authors:** Byron H. Daggett

**Affiliations:** Buffalo, N. Y.


### GENITO-URINARY AND SYPHILITIC DISEASES.

Conducted by BYRON H. DAGGETT, M. D., Buffalo, N. Y.

### SALICYLATE OF SODIUM AND URIC ACID.

BOHLAND (*British Medical Journal*, February 8, 1896,) states that it would appear, according to the researches of some authorities, and it is confirmed by his own investigations, that this drug produces an increased excretion of uric acid only when there is an increased production of this substance.

According to Horbaczewski, uric acid is the metabolic product of nuclein, which latter is derived from the white blood cells.

Hecken and Bohland found that sodium salicylate considerably increased the excretion of uric acid, but that the number of leucocytes was also greatly increased.

Medium doses of salicylates produce a distinct increase in the white cells, and, if Horbaczewski's theory is correct, a considerable leucolysis, and through this latter an augmented uric acid excretion.

Sodium salicylate has been found useless in a number of cases of gout. Some authorities admit that it is of no service in cutting short the attack, but claim that it is a valuable analgesic in gout. According to this evidence salicylates should not be used in gout or in the uric acid diathesis.

---

### THE SERUM TREATMENT OF SYPHILIS.

Borling (*British Medical Journal*, February 8, 1896,) reports a case of syphilis successfully treated by serum.

His patient was a middle-aged man, who presented a chancre on the dorsum of the penis, over the corona, with all of the characteristics of a Hunterian sore. This chancre spread and developed phagedena, growing rapidly, in spite of the usual treatment by mercurials for a month.

Antisyphilitic serum 1 c.cm. was injected December 31st into the left flank. The evening temperature was normal and there was no apparent effect.

January 1, 1896, 2 c.cm. of serum were injected into the right flank. This caused malaise for about one hour and distinct redness and increased swelling round the chancre.

January 2d, 3 c.cm. were injected in the left flank, producing no further appreciable change.

January 3d, 4 c.cm. were injected in right flank. The chancre had not spread since the beginning of the serum treatment and the patient felt "all right." Temperature slightly increased.

January 4th, the eruption had nearly disappeared and the slough was separating.

January 6th, the slough was detached and the surface was granulating. General condition of patient improving.

January 25th, the patient had gained in weight. The wound was nearly healed.

Owing to the urgency of the symptoms 10 c.cm. of serum had been given in four days. Apparently, the serum treatment was effective and prompt.

---

### CASTRATION FOR HYPERTROPHY OF THE PROSTATE.

KORSING (*British Medical and Surgical Journal*, February 8, 1896,) has changed his views regarding the value of this treatment. This author, while recognising as an undoubted fact the rapid atrophy of the enlarged prostate after removal of both testes, held that it was a priori very improbable, that in cases of complete and chronic retention the bladder would ever renew its capacity for contraction, claiming that it was useless to remove the obstruction when the bladder had lost its function for expelling the urine.

The persistency of retention in spite of regular and repeated catheterisation was, in his opinion, a conclusive indication that the function of the bladder had been hopelessly abolished.

These conclusions were upset by the following case: A man 85 years of age, who had not passed a drop of urine, except by artificial means, for eleven years, was able to pass water normally two months after castration, the residuum at the end of twenty-four hours amounting to 30 c.cm.

The operation was performed for the purpose of facilitating the passage of the catheter. The occurrence of complete retention in cases of enlarged prostate is difficult to account for, after this recorded clinical fact, by views hitherto held by surgeons. The restoration of the contractility of the bladder after eleven years, opposes the views of Guyon on coördinate arterio-sclerosis and the purely mechanical explanation of hypertrophy, hyper-distention, atrophy and paralysis. Eleven years of catheterisation, according to our accepted theories, would irretrievably destroy the contractile function of the bladder.

Whether retention is due to the increased size or form of the diseased prostate, or whether the loss of vesical contractility and prostatic hypertrophy are the results of a single and as yet unknown cause, are open questions.

---

ENURESIS.

Nason (*British Medical Journal*, February 8, 1896,) reports a case of nocturnal incontinence of twenty-two years' standing cured by galvanism :

S. H., female, 22 years of age, had suffered from her earliest childhood with enuresis and too frequent micturition. Her health was good in all other respects and the urine apparently normal. Neither belladonna, rhus aromatica nor other remedies had any influence in relieving her malady.

Nason then applied the following procedure with success : He passed a No. 10 catheter into the bladder, attaching it to an irrigating fountain containing a solution of boric acid, which was injected by slight force of gravity, until slight pain was evident, or the bladder was comfortably full, when the positive pole of a constant current battery was applied over the lumbar spine and the negative electrode was placed in the reservoir. A weak galvanic current was passed for ten minutes. This process was repeated every night for a fortnight and then at longer intervals. The toleration of the bladder was increased at the end of a fortnight so that it would hold twenty ounces, beginning with four. This treatment was continued fortnightly for some months and the patient remained practically well four months after having been discharged convalescent.

A boy, who for five years, night and day, suffered from enuresis, consulted the abstracter. There was a history of injury preceding his urinary difficulty, which had been treated during this time by various remedies and posthectomy had been performed, still the malady remained unimproved and uncured.

The bulbous bougie revealed a fine resilient stricture about midway in the penile urethra. Stretching this stricture with a steel sound relieves the enuresis for longer periods each time. Cure of the stricture will undoubtedly relieve the urinary difficulty.

This stricture reflexively disturbed coördination of the urinary mechanism, inducing frequent involuntary urination.

---

### THE DIAGNOSIS OF LIQUID AND SEMI-LIQUID FORMATIONS IN THE INGUINO-SCROTAL REGION.

Manley (*Journal of Cutaneous and Genito-Urinary Diseases*, July, 1896,) denies that the diagnosis of hydrocele is usually easy, alleging that his experience with more than fifty cases of neoplastic and adventitious formations within the scrotum, during the past ten years, has convinced him decidedly to the contrary. The correct diagnosis of scrotal hygromata is a matter of greater importance than is generally supposed. Precise and accurate diagnosis points the way to simple, safe and radical treatment. For example, a serous cyst of extravaginal growth is permanently cured by decortication and avulsion. In one-fourth of his cases cystic tumors were found and removed.

*Symptomatology.*—The subjective symptoms give a hint as to the probable character of scrotal cystic tumors. Note whether a marked cachexia be present and whether there is vesical or intestinal disturbance. Impairment of sexual power indicates pressure upon the cord or testicle. Severe pain and hectic indicate tubercular disease. All tumor formations, excepting malignant and syphilitic varicocele, diminish in volume in the recumbent position. This is notable in hernia, and general in hydrocele and funicular cysts. Hydroceles and serous cysts produce no positive subjective symptoms until they attain considerable volume.

The diagnostic features of hydrocele and cerocystic disease are made manifest by: (1) increase in volume of scrotum; (2) palpation; (3) fluctuation; (4) transparency; (5) aspiration; (6) open incision.

Enlargement of the scrotum points to hydrocele, if it begins at the base, comes on gradually unaccompanied with tenderness or fever. Scrotal tumor appears suddenly, beginning in the groin. Preceded by strain and accompanied with pain, hernia is indicated. Those suffering from bubo often claim strain as a cause.

*Palpation.*—By palpation we learn the consistence, form, relations, outline, sensibility and temperature of the parts. In large or tense hydroceles the position of the testicle cannot be defined. It apparently floats in the fluid, anchored from its stalk in the gubernaculum. Again, thickening and induration of its investments renders its detection impossible or very difficult.

If we find the testicle knoblike, isolated and projected against any part of the wall of the scrotum, we may be quite sure that the tunica vaginalis is not involved, and that we have a hernia, a scrotal cyst or a neoplasm advancing downward, either from the epididymis or the funicular process.

*Fluctuation.*—The investing capsule may be distended to such a degree of resistance and hardness that fluctuation is suppressed. Again, lipomata and sarcocele may give a sense of fluctuation.

*Translucency and Transparency.*—All hydroceles do not transmit light. When fluid is present in small quantities, or when the prevaginal fascia is greatly thickened by chronic inflammation, or when pigmentation has taken place, this sign may be entirely absent. Percival Pott said that

> The transparency is the most fallible and uncertain sign belonging to it, and is a circumstance which does not depend on the quality, consistency or color of the fluid constituting the disease, so much as the uncertain thickness of the bag and of the common membrane of the scrotum. If they are thin, the fluid is so quickly formed as not to give the tunica vaginalis time to thicken much ; the ray of light may be seen to pass through the tumor, but this is accidental and is not to be depended upon. Whoever would be acquainted with the disease must learn to distinguish it by other and more reliable means, or he will be apt to fall into disgraceful and pernicious blunders.

The scrotal distension of general dropsy is always transparent to reflected light. Evidently transparency and fluctuation are not, of themselves, positive and certain in differential diagnosis of scrotal tumors.

*Puncture or Aspiration.*—It decides the presence or absence of fluid. If fluid be withdrawn, its character and source may be determined. Before puncture the question of hernia must be eliminated. The fluid of hydrocele presents many of the characters of plasms and not that of an aqueous transudation. Its characteristics point to inflammatory origin. The microscope shows fragments of endothelia, a granular substance, cholesterine and, occasionally, spermatozoa.

*The Open Incision.*—This is more positive and exact than all other means of diagnosis combined. The free, open incision permits thorough exploration of the parts by the senses of sight and touch, making diagnosis positive and at the same time it is effective as a cure. Under complete antiseptic precautions exploratory incision is justifiable as a diagnostic expedient; at the same time it opens the way for a rapid, complete and successful surgical procedure. Scrotal vitality is feeble, hence this incision should not be practised upon debilitated old men.

*The Diagnosis of Complicated Hydrocele.*—Hydroceles occasionally complicate hernia. While their anatomical bases and physical characters widely differ, yet they are alike in one respect: they form scrotal tumors. Accumulations of fluid within the scrotum seldom cause inconvenience, except they attain considerable volume.

In all cases of hernia complicated with sufficient effusion to cause inconvenience or suffering, when there are no contra-indicating conditions present, we should not hesitate to open, evacuate and inspect. Nonreducible hernia, in children, complicated with cyst or hydrocele, incision will render diagnosis certain and favor spontaneous cure.

Victor Bougu (*Thèse de la Faculté de Paris*, 1896,) emphasises the advantage of the free incision as a precise means of diagnosis in all varieties of cystic diseases of the scrotum. Absolute diagnosis determines rational, definite treatment. About one-third of the cases sent to Dr. Manley for treatment of supposed hydrocele were proven by incision to be purely neoplastic or adventitious formations. They presented the general features of hydrocele, differing in their mode of development and in the position of the testis. In large hydroceles the testis cannot be detected, while in scrotal cysts it is isolated, and, occupying an independent position, some of these hygromata behave much like hernia. Uncomplicated neoplastic cysts develop within the loose myxomatous tissue of the spermatic cord, or from the "hydatid of Giraldes." There are no constitutional disturbances, no history of infection, no fever, hectic or pain. Their fluid contents shed some light on their pathological composition. It is of lower specific gravity and has fewer epithelial cells than the fluid of hydrocele. It is watery, colorless and alkaline in reaction and occasionally a few disintegrated spermatozooids are found. In doubtful cases, make a free incision for positive diagnosis as well as for a radical cure by dissection. All danger is avoided by thorough asepsis and hemostasis.

